# Analysis of Post-operative Adjuvant Chemotherapy Versus Adjuvant Chemoradiation Therapy Outcomes in Non-metastatic Cholangiocarcinoma: an NCDB Review

**DOI:** 10.1007/s12029-021-00696-w

**Published:** 2021-09-06

**Authors:** Robin R. Rodriguez, Stephen Abel, Jyothika Mamadgi, Paul B. Renz, Rodney E. Wegner, Moses S. Raj

**Affiliations:** 1 Department of Medical Oncology, Allegheny Health Network Cancer Institute, Allegheny General Hospital, Level 01, 320 E. North Avenue, Pittsburgh, PA 15212 USA; 2 Department of Radiation Oncology, Allegheny Health Network, Cancer Institute Pittsburgh, Pa, USA

**Keywords:** Cholangiocarcinoma, Adjuvant treatment, Chemotherapy, Radiation therapy

## Abstract

**Background:**

Each year, approximately 8000 cases of cholangiocarcinoma are recorded in the USA. Surgical resection is considered to be the only curative option. Despite surgery as a curative approach, many patients will require adjuvant therapies in the form of chemotherapy (ChT) or chemoradiotherapy (CRT). As such, we sought to analyze outcomes in patients with non-metastatic cholangiocarcinoma receiving adjuvant ChT or CRT following surgical resection.

**Methods:**

We queried the National Cancer Database (NCDB) for patients with a diagnosis of non-metastatic cholangiocarcinoma between the years 2010 and 2015 who underwent adjuvant ChT or CRT following surgery. Overall survival (OS) was calculated using Kaplan Meier method. Cox proportional hazard ratios were used to identify predictors of overall survival, and logistic regression was used to identify predictors of receiving each treatment.

**Results:**

A total of 875 patients were identified who met the above eligibility criteria. Of these patients, 818 received adjuvant chemotherapy alone with 57 patients receiving adjuvant chemoradiation therapy. The median OS in patients receiving CRT was 19.8 months versus 11.9 months for ChT (*p* value < 0.0238). The 1- and 5-year survival rates between ChT and CRT were 50% vs 61% and 6% vs 13%, respectively (hazard ratio 0.7005; 95% CI 0.51–0.97; *p* value < 0.0294).

**Conclusion:**

The results of this study suggest a potential benefit of chemoradiation therapy in the adjuvant setting, although the trends appear to show rare utilization. Given the limitations of our study, prospective corroboration is warranted.

## Introduction

With an incidence of approximately 8000 cases annually in the USA, cholangiocarcinoma is a rare form of cancer [1]. Recent studies are beginning to demonstrate the complex nature of this disease, with important implications stemming from its molecular pathogenesis [2]. This heterogeneous disease derives from the epithelium and is categorized according to its anatomic location as either intrahepatic or extrahepatic [2, 3]. Each of these types presents unique challenges in treatment and management due to their different epidemiology and prognosis [4]. At present, surgical resection is the only potential curative option [3]. This option however is not available for many patients considering that the disease is either locally advanced or metastatic at diagnosis [5]. Even with curative resection, many patients require adjuvant therapies in the form of chemotherapy (ChT) alone or chemoradiotherapy (CRT) [6]. Due to the risk of recurrence, it is recommended that patients are either enrolled in clinical trials or begun on adjuvant therapy [6]. To indicate the rationale for the use of adjuvant therapy in post-operative patients, a phase III clinical trial demonstrated an improvement in overall survival in patients receiving adjuvant chemotherapy versus surgery alone [7]. Benefits reported with the use of adjuvant chemoradiation have been sparse, with many studies being interpreted with mixed results [8]. As such, we sought to analyze survival outcomes and identify variables predictive of adjuvant ChT or CRT receipt in patients with non-metastatic cholangiocarcinoma using the National Cancer Database (NCDB).

## Methods

The NCDB is a joint program which is managed by both the American Cancer Society and the American College of Surgeons [9]. This oncological database represents approximately 70% of cancer cases in the USA annually and extrapolates its data from over 1500 Commission on Cancer (CoC)–accredited facilities [9]. As the information contained within the database is de-identified, this study was exempt from institutional board review (IRB) supervision. The results and analysis included herein have not been verified by either the American Cancer Society or the American College of Surgeons; and these programs do not take responsibility for the conclusions that result from this study. As this methodology has been undertaken in previous studies, a similar analysis has been done to extrapolate these results [10, 11].

**Fig. 1:**
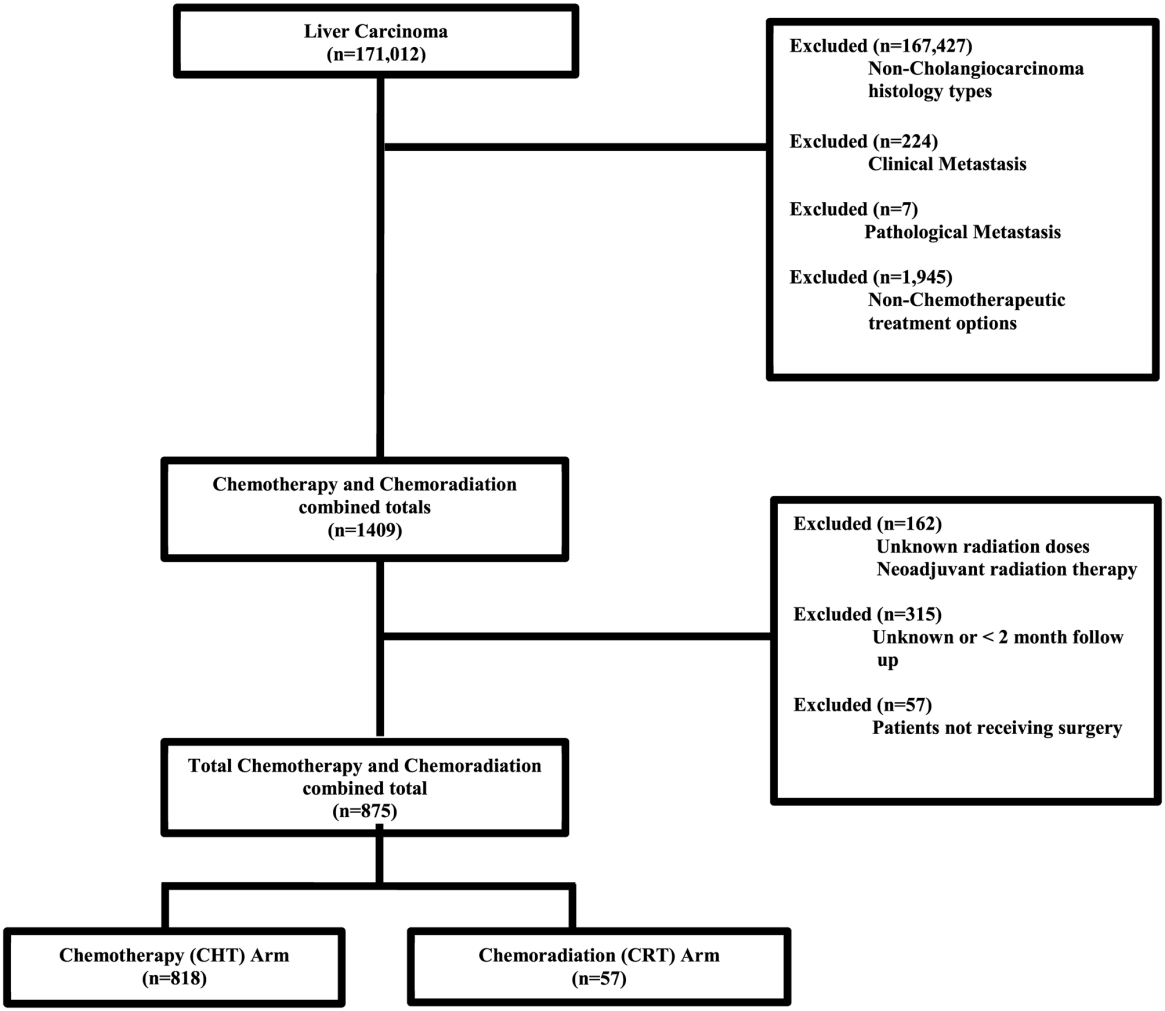
CONSORT Diagram. Chemotherapy vs chemoradiation therapy

Within this study, we utilized the NCDB liver database from 2010 to 2015. Cholangiocarcinoma, adenocarcinoma, and Klatskin tumor of histology codes 8140/8160/8162, respectively, were used [12, 13]. Inclusion criteria were all cases that were clinically and pathologically non-metastatic and those which received surgery as their first treatment option. Patients who did not receive either postoperative chemotherapy or chemoradiation therapy were excluded along with those patients who had less than 2 months follow-up to account for immortal time bias. A CONSORT diagram that depicts the inclusion criteria is shown in Fig. 1. Utilizing the exclusion criteria, a total of 875 patients were eligible for final analysis. Of these 875 patients, 818 were found to have received chemotherapy alone, whilst 57 patients received both chemotherapy and radiation therapy. The information that was analyzed from the database on the patients included clinical, treatment, and baseline characteristics. Race was defined as either white, African American, or other/unknown. Data was obtained from this information by the performance of statistical analysis via MedCalc Version 18 (Ostend, Belgium).

The results were reported from the use of both univariable and multivariable logistic regression models used to determine associated parameters of interest. The overall survival was calculated using the date of diagnosis and the date of last contact or time of death with a Kaplan–Meier curve. Univariable survival analysis was carried out for all characteristics as listed in Table 1. Following, statistically significant parameters were then used to determine adjusted hazard ratios (HR) along with a 95% confidence interval (CI) calculated with a *p* value of 0.05. A Cox proportional hazards model was developed to assess relation of multivariable parameters.

**Table 1:** Patient and treatment characteristics (*N* = 15,110)

Characteristic	No. (% or range)	Characteristic	No. (% or range)
**Demographics**		Treatment facility type	
		Community cancer program	483 (3.2)
Sex		Academic/research program	6570 (43.5)
Male	6892 (46)	Comprehensive cancer program/other	8057 (53.3)
Female	8218 (54)	Rural counties	339 (2.3)
Age		Year of treatment	
Median	75 (40–90)	2004–2007	526 (3.5)
≤ 65	2424 (16.0)	2008–2011	4252 (28.1)
> 65	12,686 (84.0)	2012–2015	10,332 (68.4)
Race		**Disease characteristics**	
White	13,617 (90.1)		
African American	1190 (7.9)	Clinical T stage	
Other/unknown	303 (2.0)	T1	11,845 (78.4)
Comorbidity score		T2	3,265 (21.6)
0	8558 (56.6)	Histology	
1	4031 (26.7)	Adenocarcinoma	8924 (59.1)
2 +	2521 (16.7)	Squamous Cell Carcinoma	6186 (40.9)
Insurance		Grade	
Private	1869 (12.4)	Well differentiated	1453 (9.6)
Government	12,951 (85.7)	Moderately differentiated	3033 (20.1)
Unknown	180 (1.2)	Poorly differentiated	2833 (18.8)
Education		Unknown	7791 (51.6)
≥ 29	2113 (14.0)	**Treatment characteristics**	
20 to 28.9	4138 (27.4)	Radiation dose, Gy	
14 to 19.9	5378 (35.6)	Median (range)	
< 14	3435(22.7)		50.0 (30.0–75.0)
Unknown	54 (0.3)	Interquartile range (Gy)	
Income, US dollars			5.5
< 30,000	2685 (17.8)	Fractionation	
30,000 to 35,000	3890 (25.7)	Median (fraction number)	
35,000 to 45,999	4343 (28.7)		4(1–5)
≥ 46,000	4132 (27.4)	Biologically equivalent dose, Gy10	
Unknown	60 (0.4)	Median (Range)	
Distance to treatment facility, miles			112.5 (100–231.9)
< 10	7046 (46.6)	Interquartile Range (Gy10)	
> 10	8064 (53.3)		51.2

## Results

**Fig. 2:**
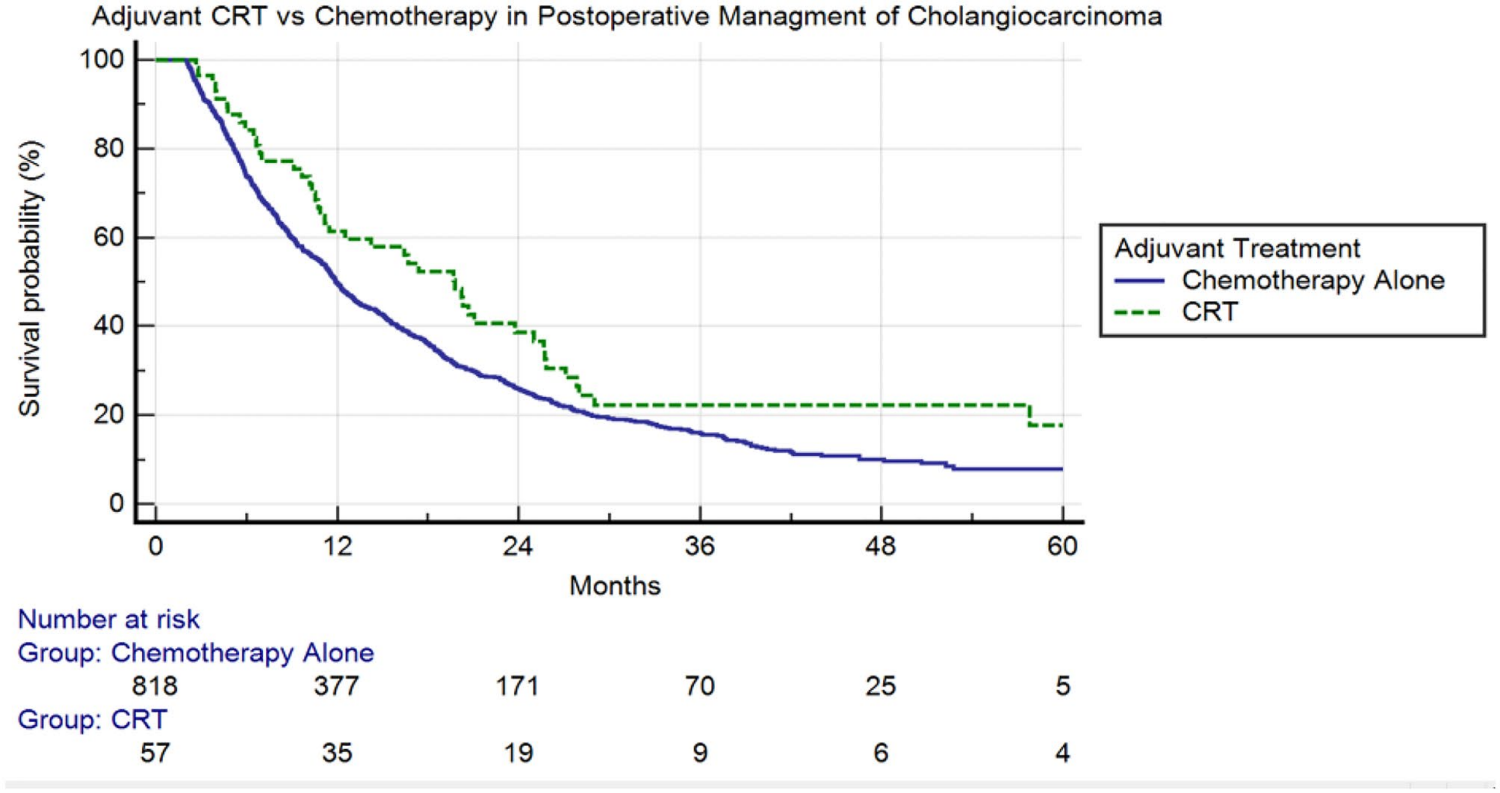
Kaplan Meier analysis

From the NCDB database between the years of 2010 and 2015, a total of 875 patients with non-metastatic cholangiocarcinoma treated with either adjuvant chemotherapy or adjuvant chemoradiotherapy following surgery were eligible for assessment. From these patients, the baseline characteristics are demonstrated in Table 1. Median age was 62 years. The majority of patients were white in race (87%), and there was a small predominance of female over male patients (51% and 49%, respectively). Although the data collected was from 2010 to 2015, no cases of cholangiocarcinoma were able to be included from 2015 due to our exclusion criteria. Thus, only cases from the years 2010–2014 were included in this study. The majority of the cases (66%) took place between 2012 and 2014. Table 2 details differences within demographic and disease-related characteristics between those who received chemotherapy versus chemoradiotherapy. Statistical significance was determined for two variables. Patients were more likely to obtain chemoradiation if they had positive surgical margins (*p* < 0.01). In addition, patients receiving chemoradiation were more likely to live closer to the treatment facility (*p* < 0.02). Using Kaplan Meier analysis, the median overall survival was calculated for both cohorts. Median OS was 11.9 months for ChT and 19.8 months for CRT. At 1 year, 3 years, and 5 years, the OS for ChT was 50%, 16%, and 6% versus CRT at 61%, 22%, and 13%, respectively (*p* < 0.02). These results can be seen in Fig. 2. Median follow-up collectively for all cases was determined to be 11.4 months. The interquartile range for follow-up was 5.7–21.4. On multivariable analysis, it was determined that improved OS was associated with CRT, female gender, lower co-morbidity score, and race other than white or African American (Table 3).

**Table 2:** Comparative analysis of adjuvant chemotherapy versus adjuvant chemoradiation therapy by baseline characteristics in non-metastatic cholangiocarcinoma cases

Characteristic	Chemotherapy (ChT) (*n* = 818) (%)	Chemoradiation therapy (CRT) (*n* = 57) (%)	Odds ratio	95% CI	*p*
Sex					
Male	395 (48)	32 (56)	1	Ref	
Female	423 (52)	25 (44)	0.80	0.44–1.47	0.46
Race					
White	708 (87)	53 (94)	1	Ref	
African American	62 (7)	2 (3)	0.69	0.15–3.14	0.63
Other	48 (5)	2 (3)	0.83	0.18–3.82	0.81
Comorbidity score					
0	557 (68)	43 (75)	1	Ref	
1	184 (23)	12 (21)	0.79	0.38–1.64	0.52
≥ 2	77 (9)	2 (4)	0.34	0.08–1.49	0.15
Age					
> 65	435 (53)	28 (49)	1	Ref	
< 65	383 (47)	29 (51)	1.028	0.50–2.13	0.94
Insurance					
None	25 (3)	1 (1)	1	Ref	
Private payer	326 (40)	29 (52)	2.76	0.31–24.51	0.36
Government Unknown	458 (56) 9 (1)	26 (46) 1 (1)	1.63 4.76	0.18–14.45 0.22–102.29	0.66 0.32
Facility type					
Community Cancer Program/Comprehensive Cancer	419 (51)	21 (37)	1	Ref	
Academic Research	315 (39)	29 (51)	1.32	0.67–2.58	0.42
Integrated Cancer Network	84 (10)	7 (12)	2.05	0.77–5.46	0.15
Income, USD					
< 30,000	133 (16)	12 (21)	1	Ref	
30,000–35,000	216 (26)	9 (16)	0.36	0.13–1.04	0.06
35,000–45,999	228 (28)	15 (26)	0.65	0.23–1.80	0.40
> 46,000	241 (30)	21 (37)	0.70	0.23–2.16	0.53
Education					
≥ 29%	146 (18)	9 (16)	1	Ref	
20 to 28.9	221 (27)	15 (26)	1.06	0.40–2.81	0.90
14 to 19.9	264 (32)	18 (32)	1.22	0.41–3.61	0.72
< 14	187 (23)	15 (26)	1.24	0.37–4.18	0.73
T stage					
1	44 (5)	6 (10)	1	Ref	
2 3 4 Unknown	40 (5) 20 (2) 9 (1) 705 (87)	9 (16) 0 (0) 2 (4) 40 (70)	1.77 2.32^E009^ 2.23 0.37	0.49–6.44 0.31–16.04 0.08–1.71	0.39 1.00 0.42 0.20
Surgical margins					
No	60 (7)	9 (16)	1	Ref	
Yes Unknown	13 (2) 745 (91)	5 (9) 43 (75)	6.38 0.63	1.46–27.85 0.25–1.55	** < 0.01** 0.31
Distance to facility					
> 11 miles	383 (47)	38 (67)	1	Ref	
< 11 miles	435 (53)	19 (33)	0.84	0.78–0.89	** < 0.02**

Education is quartiles of the percentage of persons with less than a high school education in the patients’ residence census tract. Income is median household income in the patients’ residence census tract

**Table 3:** Multivariable Cox proportional hazards models for overall survival in patients with resected non-metastatic cholangiocarcinoma receiving adjuvant chemotherapy versus chemoradiotherapy

Significant characteristic	Hazard of death (95% CI)	*p*
	Cox model	
Age		
> 65	Reference	
< 65	1.02 (0.84–1.23)	0.86
Sex		
Male	Reference	
Female	0.79 (0.68–0.92)	** < 0.003**
Insurance		
None	Reference	
Government Private	1.16 (0.72–1.88) 1.00 (0.62–1.60)	0.53 0.99
Unknown	1.07 (0.44–2.60)	0.89
Comorbidity score		
0	Reference	
1	0.92 (0.76–1.11)	0.37
≥ 2	1.40 (1.07–1.83)	** < 0.01**
Race		
White	Reference	
African American	1.07 (0.79–1.43)	0.67
Other	0.61 (0.42–0.88)	** < 0.008**
Income		
< 38,000	Reference	
38,000–47,999	1.09 (0.84–1.42)	0.51
48,000–62,999	0.97 (0.73–1.28)	0.82
> 63,000	0.94 (0.70–1.60)	0.71
Facility type		
Community Cancer Program/Comprehensive	Reference	
Academic/Research Program	0.95 (0.80–1.13)	0.56
Integrated Network Cancer	0.87 (0.66–1.12)	0.28
Education		
≥ 29%	Reference	
20 to 28.9	1.15 (0.89–1.48)	0.27
14 to 19.9	1.12 (0.85–1.47)	0.43
< 14	1.30 (0.95–1.77)	0.10
Radiation treatment		
Chemotherapy	Reference	
Chemoradiotherapy	0.70 (0.51–0.97)	** < 0.03**

Education is quartiles of the percentage of persons with less than a high school education in the patients’ residence census tract. Income is median household income in the patients’ residence census tract

## Discussion

Cholangiocarcinoma, a rare biliary tract cancer, is a heterogeneous disease with an aggressive natural history [2]. In addition to anatomic location, surgical margins are known prognostic factors with R0 resected margins being shown to have clinically better outcomes and improved 5-year OS [14, 15]. Despite surgical resection being a potentially curative option, a very limited number of patients with cholangiocarcinoma are eligible for surgical resection [4]. Additionally, those with surgical resection still have the potential for recurrence. A retrospective study involving patients with intrahepatic cholangiocarcinoma demonstrated 1-year, 2-year, and 3-year recurrence-free rates of only 16.2%, 5.4%, and 2.7%, respectively [16]. Corroborating this, a large case series of 920 patients treated with surgical resection for intrahepatic cholangiocarcinoma demonstrated 607 patients (66%) who developed recurrence of the disease [17]. Comparatively, in surgically resected perihilar and distal extrahepatic cholangiocarcinoma, the recurrence rate ranged from 60 to 75% [18]. Poor prognosis of this disease and the risk of recurrence following surgical resection in local stages illustrate the imperative need to consider adjuvant options for management. Chemotherapy and chemoradiation therapy are two of the main adjuvant options offered to non-metastatic cholangiocarcinoma patients following surgical resection. Current clinical trials studying the effects of these options include the Adjuvant Chemotherapy with Gemcitabine and Cisplatin Compared to Standard of Care After Curative Intent Resection of Biliary Tract Cancer (ACTICCA-1 trial) [19]. The results of this trial are currently still pending [19]. Continued studies such as ACTICCA-1 were initiated due to the encouraging findings of the BILCAP (capecitabine compared with observation in resected biliary tract cancer) trial [20]. This phase III randomized, multicenter study analyzed 447 patients, 223 of which were given capecitabine following surgical resection [20]. The results found that the recurrence-free survival for the capecitabine group was 24.4 months (95% CI, 19.8–46.3) versus 17.5 months in the control group (95% CI, 12.0–23.8) [20]. Aside from capecitabine, the European Study Group for Pancreatic Cancer (ESPAC-3) trial also demonstrated efficacy of both gemcitabine and fluorouracil with a median OS of 43.1 months (95% CI, 34.0–56.0) for the two chemotherapy groups versus 35.2 months (95% CI, 27.2–43.0) for the control group [21]. Although the ESPAC-3 focused on periampullary cancers, it is understood that cholangiocarcinoma cases are often included in studies of periampullary disease [6]. Another phase III trial conducted by investigators in the UK assessing the role of chemotherapy in biliary tract cancers also provided insight into multi versus single agent therapy use [22]. Within this study, 410 patients with locally advanced or metastatic cholangiocarcinoma and other biliary tract cancers were assessed [22]. The cohort arms were divided into either cisplatin followed by gemcitabine vs gemcitabine alone [22]. The study identified an increased median OS and progression-free survival in the combination chemotherapy group vs the single-agent therapy group (11.7 months/8 months vs 8.1 months/5 months) [22]. The aforementioned study largely lends to the importance of considering multi agent therapies and corroborates the efficacious findings of chemotherapy in management of cholangiocarcinoma.

The use of chemoradiation therapy in the adjuvant setting, unlike chemotherapy alone, has demonstrated more heterogeneous results. Of note, chemoradiation therapy is often used in adjuvant management of patients with positive R1 resection margins [6]. This is similar to findings within our study that CRT was utilized more in patients with positive margins. Illustrating this, one retrospective review studied patients with R0 margins treated with surgery-only versus R1 margin patients treated with adjuvant CRT post-operatively [8]. The results demonstrated similar OS between the two groups (42% versus 36%, *p* < 0.6) [8]. On the other hand, contrary to the aforementioned findings, a phase III trial conducted by the European Organization of Cancer Research (EORTC) did not demonstrate oncologic benefit with the addition of adjuvant chemoradiation [23]. A limitation of this study was that the cholangiocarcinoma arm was relatively small. [23].

In comparing differing adjuvant therapies and their effect on survival, previous research has been conducted. One such study reviewed outcomes of 599 patients who were administered adjuvant therapy following surgical resection in intrahepatic cholangiocarcinoma cases [24]. These results illustrated improved survival with CRT versus ChT alone [24]. Overall, the results of our study stand to corroborate the findings of Lin et al. in that, patient receiving CRT had improved OS versus ChT alone [24]. Within the trial of Lin et al., the 2-year OS between concurrent chemoradiotherapy and chemotherapy alone was 48% versus 38% [24]. In comparison of these findings in conjunction with our study, the results for overall survival are similar. It is important to note that although 3–5-year survival data is often of particular interest, both our study and that of Lin et al., the 1- and 2-year survival OS was significant to address as the median OS of cholangiocarcinoma in and of itself is relatively short [24]. A limitation of our study in this regard however is the lack of separation between concurrent and sequential radiotherapy. Another study assessed the NCDB database for the results of differing adjuvant therapies [25]. This study looked at patients from the years 1998–2006 who were treated for extrahepatic cholangiocarcinoma [25]. This study by Hoehn et al. had a large sample population with a total of 8741 patients [25]. Within their study, 3 arms where identified: surgery alone, adjuvant ChT, or CRT following surgery [25]. From these groups, the patients given adjuvant CRT were noted to have increased OS versus those who had surgery only or adjuvant ChT [25]. Additionally, and similar to our study, Hoehn et al. also concluded that females were more likely to have better outcomes than males [25]. Other important results from their study were the corroboration that worse survival was associated with positive margins along with advanced stage of disease [25]. Another NCDB review of extrahepatic cholangiocarcinoma from the years 2004–2014 also noted increased survival with CRT adjuvant therapy [26]. However, this increased survival with the use of CRT versus ChT was only noted in patients with positive resection margins only [26]. Without the positive margins, OS was similar between the ChT and CRT groups at a total of 36 months [26]. Although the aforementioned study along with others demonstrated the significance of surgical margins in relation to OS, our study was not able to corroborate that margins were predictive of survival due in part to our small sample size [25, 26]. In regard to studies comparing adjuvant therapies in intrahepatic cholangiocarcinoma, a study done of the NCDB from the years 1998–2006 revealed that there was increased survival in patients with positive resection margins who received either type of adjuvant therapy (CRT or ChT) [27]. In this study, it was concluded that there was no significant improvement in survival with adjuvant therapy if the patients were node negative [27]. In this study, analysis of ChT versus CRT was not conducted; so, no conclusions can be drawn as to whether one type of adjuvant therapy conferred increased survival benefit over the other [27].

Ultimately, the results of our study provide further evidence for the need of continued clinical trials to validate the use of adjuvant therapies in patients with cholangiocarcinoma. Although multiple studies have been undertaken to assess the benefit of adjuvant therapy in post-resected cholangiocarcinoma cases, there is still no consensus as to which type of adjuvant therapy (CRT or ChT) is more beneficial in relation to overall survival. Thus, further research should be directed towards determining OS between chemoradiation therapy versus chemotherapy alone as mounting evidence inclusive of the results of our study are illustrating that CRT versus ChT alone is associated with improved OS. Although our study is not the first to be done in reporting results of cholangiocarcinoma cases, the use of the NCDB offers advantages over single institution cohorts alone. Consequently, the information contained therein can be applied to a greater population of cholangiocarcinoma patients. Additionally, our report is one of a few studies that focuses solely on non-metastatic cases. This is important in that it is evident that even in local stages, adjuvant therapy needs to be a consideration for continued management of this disease due to the potential for recurrence as previously mentioned [6, 16]. Despite the aforementioned results of CRT associated with improved OS, it is important to note that our study is not without limitations. These limitations are inclusive of selection bias as the patient data was obtained via NCDB, inability to distinguish intrahepatic versus extrahepatic disease, inability to report patient functional status asides from comorbidity score, along with unknown rates of local or distant control. Additionally, despite statistically significant findings in relation to surgical margins, the majority of margins were unknown as well as the majority of T staging. Other limitations revolve around unknown variables surrounding chemotherapy and chemoradiotherapy such as unknown chemotherapy agents, unknown duration, unknown toxicity profile, and unknown number of cycles. Our criterion was also limited to non-metastatic cases. Lastly and most potentially the largest limitation of our study is the relatively small sample size of our chemoradiation group. Consequently, retrospective studies should be further corroborated by phase III trials which can properly randomize and diminish confounding variables.

## Conclusion

Overall, the scope of our study was to review adjuvant treatment options and their outcomes for post-operative patients with non-metastatic cholangiocarcinoma. The results suggest a potential benefit of chemoradiation therapy in the adjuvant setting over chemotherapy alone; although the trends appear to show rare utilization. Given the limitations of our study, prospective corroboration is warranted.

This study has not been presented or published in part or full in any other form elsewhere.

## Data Availability

The information that was used to produce the resulting graphs and statistical analysis was derived from the National Cancer Database (NCDB).

## Declarations

### Checklist

The authors have completed the STROBE reporting checklist.
